# Patient-centred orientation among healthcare professionals in a Singapore community hospital

**DOI:** 10.1186/s12913-026-15252-0

**Published:** 2026-07-27

**Authors:** Xiao-Ying Cleo Chiong, Lay Hoon Goh, Anna Szücs, Wei Mon Wong

**Affiliations:** 1 National Healthcare Group Polyclinics, 1 Mandalay Road, Singapore, 308205 Singapore; 2 National University Polyclinics, 1 Jurong East Street, #21, Singapore, 609606 Singapore; 3 Faculty of Behavioural and Movement Sciences, Vrije Universiteit Amsterdam, De Boelelaan 1105, Amsterdam, 1081 HV The Netherlands; 4 St Luke’s Hospital, 2 Bukit Batok Street 11, Singapore, 659674 Singapore

**Keywords:** Burnout, Healthcare professionals, Organisational culture, Patient-centred care, Patient-centred orientation, Patient-practitioner orientation scale, Post-acute care, Subacute care

## Abstract

**Background:**

Patient-centred care (PCC) is central to high-quality healthcare. Yet few studies have examined the orientation of multidisciplinary staff toward patient-centredness in community hospital settings. The study aimed to assess patient-centred orientation among healthcare professionals in a Singaporean community hospital and to identify factors associated with this orientation.

**Methods:**

A cross-sectional survey of 261 physicians, nurses, and rehabilitation professionals was conducted in St Luke’s Hospital, a community hospital in Singapore. The 18-item Patient-Practitioner Orientation Scale (PPOS) was used to assess patient-centred orientation, with higher scores indicating greater patient-centredness. Multivariable linear regression analyses identified factors associated with patient-centred orientation.

**Results:**

The mean PPOS score was 3.75 points (*SD* = 0.63) on a 1–6 scale. Physicians scored significantly higher than nurses and therapy aides (*p* < 0.001). In multivariable analysis, professional role, a hospital culture that promotes PCC, and occasional stress without burnout were independently associated with higher PPOS scores.

**Conclusion:**

Significant variation in patient-centred orientation may exist across professional groups, highlighting the need for targeted interventions to foster patient-centred orientation, particularly among nurses and therapy aides. Organisational culture and staff well-being also represent important considerations for promoting patient-centred orientation among healthcare professionals. Further research should examine whether patient-centred orientation translates into patient-centred behaviours in clinical practice.

**Supplementary Information:**

The online version contains supplementary material available at https://doi.org/10.1186/s12913-026-15252-0.

## Background

Patient-centred care (PCC) is central to high-quality healthcare. It promotes the understanding of the patient as a whole person, respecting individual preferences and values, and involving patients actively in healthcare decision-making [1–7].

Evidence demonstrates that PCC contributes to improved health outcomes, greater patient satisfaction, adherence to treatment, and more efficient healthcare utilisation [8–13]. In Singapore, this shift is mirrored in ‘Healthier SG’ [14], a multi-year strategy designed to improve preventive care and strengthen doctor-patient relationships.

Delivering PCC depends not only on systems and policies but also on the patient-centred orientation of healthcare professionals. This orientation comprises the beliefs and attitudes that practitioners hold toward their roles and their patients, and influences the extent to which patient-centred behaviours are demonstrated in clinical practice. However, patient-centred orientation can vary among practitioners, as it is influenced by factors such as professional training [15], clinical role [16, 17], institutional culture [18], burnout [19], and experience [20]. Understanding the patient-centred orientation of healthcare professionals is thus essential for informing efforts that aim to strengthen patient-centred care.

The Patient-Practitioner Orientation Scale (PPOS) is a widely used and validated instrument to assess healthcare professionals’ orientation toward patient-centredness that was developed by Krupat et al. [20–22]. It captures two distinct domains of PCC, described as ‘Sharing’ and ‘Caring’ respectively. The PPOS ‘Sharing’ domain refers to the extent to which practitioners believe in sharing power, information, and decision-making with patients. This has its roots in the notable work of Byrne and Long [7], who characterised patient-centred physicians as those who share information and involve patients as equals in decision-making. The PPOS ‘Caring’ domain captures the degree to which practitioners value empathy, psychosocial engagement, and treating the patient as a whole person. This orientation is rooted in the work of Levenstein and Stewart [5, 6], which emphasises the importance of exploring the patient’s feelings, expectations, and the human experience of illness rather than employing a strictly biomedical approach.

Most studies using the PPOS have focused on physicians or students in academic settings, tertiary, and primary care [20–30]. However, empirical evidence from Singapore is limited, particularly regarding multidisciplinary teams in community hospitals. This represents a significant gap in evidence, as community hospitals in Singapore play a key role in delivering PCC for our ageing population with complex medical and psychosocial needs [31].

Community hospitals in Singapore serve as intermediate, post-acute care facilities for patients who have completed their acute treatment but are not medically or functionally ready to return to their homes or long-term care facilities. St Luke’s Hospital (SLH) is one such institution: a 243-bed community hospital delivering rehabilitative, subacute, and palliative care. SLH is also unique in its integration of care across the continuum; in addition to its inpatient facility, it provides outpatient and home care services that support patients transitioning back into the community. Patients admitted to SLH are typically frailer and more complex with multiple medical, functional, and psychosocial needs [31]. For these reasons, it requires sustained collaboration between all members of the healthcare team, and with the patient, to deliver quality care. Consequently, having a patient-centred orientation among the healthcare team is fundamental to providing holistic and quality care.

Within this context, this study aimed to evaluate the extent of patient-centred orientation among multidisciplinary healthcare professionals at SLH. The primary objectives were to determine the baseline orientation of healthcare professionals and to identify the demographic, occupational, and psychosocial factors associated with patient-centred orientation.

## Methods

### Study design and setting

This was a cross-sectional survey-based study that was conducted in SLH among employed healthcare professionals. Participation was voluntary and anonymous; informed consent was implied through survey completion.

### Participants and recruitment

Physicians, nurses, and rehabilitation therapists aged 21 years and older, who were permanent staff employed at the institution, were eligible to participate. We excluded non-permanent staff, such as locum healthcare workers, interns, and external agency personnel, as they may not be fully integrated into the hospital’s organisational culture. Staff were invited via institutional email and staff communication platforms to complete an online survey between September and November 2024.

### Assessments

#### Patient-centred orientation

Patient-centred orientation was measured using the internationally validated [21, 24–26] 18-item Patient-Practitioner Orientation Scale (PPOS) [20, 21, 32]. This scale assesses two domains: *Sharing*, which reflects beliefs about sharing information, power, and control with patients in decision-making; and *Caring*, which reflects the importance placed on empathy, good interpersonal relations, and treating the patient as a whole person. Items are rated on a 6-point Likert scale ranging from ‘Strongly disagree’ (score 1) to ‘Strongly agree’ (score 6). The overall PPOS score is the mean score of all 18 items. Higher scores indicate stronger patient-centred orientation.

We used an adapted version of the PPOS previously applied to nurses, therapists, and doctors in Singapore [33]. The internal consistency and factor structure of the PPOS were examined prior to the main analysis (Results, Psychometric Performance of the PPOS Scale). Given that these tests indicated a lack of support for a two-factor structure, a weak overall factor solution, several scale items with high uniqueness, and a higher internal consistency for the total score (Cronbach’s α = 0.794) than for either subscale (α = 0.613 and 0.704, respectively), the PPOS was treated as a unidimensional composite measure providing a global index of patient-centred orientation.

#### Independent variables

Demographic, professional, and psychosocial factors hypothesised to influence patient-centred orientation were also included in the survey.

##### Sociodemographic characteristics

Age, gender, and highest level of education attained. These were collected as they have been shown to influence patient-centred orientation in previous cohorts [25, 34, 35].

##### Professional characteristics

Clinical role (physician, nurse, therapist, or therapy aide), years of healthcare experience, full- or part-time employment status, average number of patients seen per day, usual weekly working hours, and the patient populations that participants spent the majority of time serving (e.g., palliative, dementia, wound care).

The number of patients seen per day was obtained through participant self-report. Specifically, participants were asked an open-ended question: ‘How many patients do you see on an average day?’, to which responses were restricted to numeric values in the online form. These data were collected and analysed as a continuous variable to represent daily clinical volume.

These professional characteristics were chosen because clinical role [36], healthcare experience [20, 35], workload [34], and clinical specialties [16, 27] are documented determinants of PPOS scores. Since clinical specialty is known to affect patient-centred orientation, it was of interest to evaluate whether these associations extend to staff who spend a majority of their time serving patient populations with specialised clinical needs.

##### Workplace experience and perception

Self-rated perceptions of institutional support for PCC, frequency of encountering language or cultural barriers, perceived support from colleagues/supervisors in difficult patient interactions, sense of job meaningfulness, and workload manageability (see Table 1 for a summary of the variables and their measurement characteristics).

These variables were chosen because a supportive institutional and workplace culture [37, 38], alongside peer support, and managerial support in delivering PCC [39, 40] have been shown to positively impact PCC. PCC has also been associated with perceived work meaningfulness and job satisfaction [41]. Conversely, language [42, 43] and cultural barriers [44, 45] impede care delivery, making it of interest to explore their associations with PPOS scores in our study as well.

##### Personal health and caregiving experience

Participants were asked whether they had ever received care from a healthcare professional for a medical condition, had ever been a caregiver for a family member or close friend, and whether they were experiencing current personal troubles or preoccupations.

Lived experience as a patient or caregiver was included as emerging evidence suggests that these roles can lead to a positive shift towards PCC [46, 47]. The link between personal preoccupations and troubles has not yet been established. However, it was deemed clinically relevant given that personal stressors can form a significant proportion of overall stress experienced by healthcare professionals [48], and may indirectly affect their attitudes toward patient-centred care.

**Burnout** was assessed using Dolan and colleagues’ validated single-item ordinal measure, which has been strongly associated with emotional exhaustion [49]. In this large multidisciplinary healthcare cohort (*n* = 2,285), this instrument demonstrated a very strong correlation (*r* = 0.79) and high predictive validity (*R*^*2*^ = 0.62) when compared against the Maslach Burnout Inventory (MBI) Emotional Exhaustion subscale. Specifically, this instrument assesses the full spectrum of a single domain of self-defined burnout on a five-category ordinal scale (range 1–5). Participants were asked: ‘Overall, based on your definition of burnout, how would you rate your level of burnout?’. The response options were:

1. I enjoy my work. I have no symptoms of burnout.
2. Occasionally I am under stress, and I don’t always have as much energy as I once did, but I don’t feel burned out.
3. I am definitely burning out and have one or more symptoms of burnout, such as physical and emotional exhaustion.
4. The symptoms of burnout that I’m experiencing won’t go away. I think about frustration at work a lot.
5. I feel completely burned out and often wonder if I can go on. I am at the point where I may need some changes or may need to seek some sort of help.

Burnout was chosen as a variable of interest because it has been correlated with poorer quality of care and less favourable patient-reported care experiences [50, 51].

The full survey can be found in the Supplementary material (Additional File 1, Supplementary Section S6).

### Sample size calculation

Sample size was determined based on both precision for mean estimation and power for group comparisons. Based on a previous local study which reported a mean PPOS score of 4.28 points and SD of 0.52 [33], a sample of 195 total participants was required to estimate the overall mean with a 5% margin of error and 95% confidence. For comparisons between professional groups, a power analysis was conducted. Given the relatively small headcount of healthcare professionals working at SLH (330 healthcare professionals in total, with 30 physicians, representing the smallest subpopulation), the study was constrained to detecting a large effect size (Cohen’s *d* = 0.8), as detecting smaller effect sizes would require a target sample exceeding the total headcount of physicians available in the hospital. Thus, the target was set to ensure the study was adequately powered (80% power, 95% confidence) to detect meaningful differences between professional roles, requiring a minimum of 17 participants per group. Proportional quota sampling yielded a minimum target of 249 participants comprising 17 physicians, 167 nurses, and 65 therapists.

### Pilot testing

The survey was reviewed by one physician, one nurse, and one therapist to ensure clarity and face validity.

### Statistical analysis

Descriptive statistics summarised participant characteristics and PPOS scores.

To identify independent determinants of patient-centred orientation and evaluate the relative influence of demographic, occupational, and psychosocial factors, multivariable linear regression analyses were performed. Independent variables consisted of all 18 measures included in the survey, which had been selected based on theory, clinical experience, and prior literature (see under Methods, Assessments, Independent Variables for the rationale behind each variable choice). Given the inclusion of 18 independent variables in a sample of 261 participants (approximately 14.5 observations per predictor), which could be considered low for a continuous outcome [52], a staged modelling approach was used to minimise overfitting. In the first step, each of the 18 independent variables was individually adjusted for age, gender, and education to examine their separate associations with PPOS scores (henceforth termed ‘demographic-adjusted single-variable models’; Table S4.1 to Table S4.18). In a second step, a full model (Model 2) was constructed, including all 18 independent variables simultaneously. As a last step, manual backward elimination was performed to derive a more parsimonious final model (Model 3), retaining only variables that were statistically significant in either of the initial two steps as well as basic demographic covariates, namely age, gender, and education. Model 3 was considered the principal model and used to determine the main findings. Further details of model development, selection and comparisons are provided in Supplementary Section S3 and S5.

Model assumptions were checked and adequately met. Multicollinearity was assessed via Variance Inflation Factor (VIF), with all VIF values < 2. Residuals were assessed for normality (via Q–Q plots and Shapiro–Wilk test), linearity, and homoscedasticity. The Durbin–Watson statistic confirmed independence of errors, and Cook’s distance values were all < 0.1, indicating no influential observations. All analyses were performed using Jamovi Version 2.4. A two-tailed p-value of < 0.05 was considered statistically significant.

## Results

### Sample characteristics

A total of 261 out of 330 eligible SLH healthcare professionals completed the survey, yielding a response rate of 79.1%. There were no missing data. Participants comprised 21 physicians (8.0%), 167 nursing staff (63.9%), and 73 rehabilitation therapists (28.0%). Most respondents were female (83.5%), employed full-time (93.5%) and aged between 30 and 39 years (40.6%). More than half (54.0%) held a university degree, and 36 (13.8%) held a postgraduate degree. On average, participants had 11.1 (*SD* = 8.0) years of experience in healthcare, worked 40.4 (*SD* = 13.4) hours per week and saw a mean of 15.0 (*SD* = 7.3) patients per day. Among the professional groups, nursing staff reported the highest patient contact, with an average of approximately 18 (*SD* = 6.6) patients per day. Full demographic and professional characteristics are summarised in Table 1.

**Table 1:** Demographic and professional characteristics of participants

Characteristic	Overall N (*n* = 261)	Physicians (*n* = 21)	Junior & Senior Nurses (*n* = 167)	Therapists & Therapy Aides (*n* = 73)
**Gender**				
Female	218 (83.5%)	13 (61.9%)	150 (89.8%)	55 (75.3%)
Male	43 (16.5%)	8 (38.1%)	17 (10.2%)	18 (24.7%)
**Employment status**				
Full-time	244 (93.5%)	20 (95.2%)	164 (98.2%)	60 (82.2%)
Part-time	17 (6.5%)	1 (4.8%)	3 (1.8%)	13 (17.8%)
**Age group**,** years**				
21–29	58 (22.2%)	12 (57.1%)	28 (16.8%)	18 (24.7%)
30–39	106 (40.6%)	3 (14.3%)	79 (47.3%)	24 (32.9%)
40–49	60 (23.0%)	2 (9.5%)	41 (24.6%)	17 (23.3%)
≥50	37 (14.2%)	4 (19.0%)	19 (11.4%)	14 (19.2%)
**Current role**				
Physician	21 (8.0%)	21 (100%)	0	0
Junior Nursing Staff^a^	64 (24.5%)	0	64 (38%)	0
Senior Nursing Staff^a^	103 (39.5%)	0	103 (62%)	0
Therapist	45 (17.2%)	0	0	45 (62%)
Therapy Aide	28 (10.7%)	0	0	28 (38%)
**Years in healthcare ** **(mean ± SD)**	11.1 ± 8.0	9.8 ± 11.0	12.0 ± 7.7	9.7 ± 7.4
**Hours worked per week (mean ± SD)**	40.4 ± 13.4	50.2 ± 14.4	41.0 ± 12.6	36.4 ± 13.5
**Patients seen per day** **(mean ± SD)**	15.0 ± 7.3	15.8 ± 6.2	17.8 ± 6.6	8.1 ± 3.6
**Most common types of patients served**				
Palliative patients	48 (18.4%)	4 (19.0%)	41 (24.6%)	3 (4.1%)
Dementia patients	35 (13.4%)	2 (9.5%)	24 (14.4%)	9 (12.3%)
Rehabilitation patients	131 (50.2%)	13 (61.9%)	61 (36.5%)	57 (78.1%)
Wound care patients	38 (14.6%)	0 (0.0%)	37 (22.2%)	1 (1.4%)
Others	9 (3.4%)	2 (9.5%)	4 (2.4%)	3 (4.1%)
**Perception that the hospital culture promotes PCC**				
Not at all/ To a small extent	13 (5.0%)	0 (0.0%)	8 (4.8%)	5 (6.8%)
To some extent	64 (24.5%)	3 (14.3%)	41 (24.6%)	20 (27.4%)
To a great extent	132 (50.6%)	15 (71.4%)	78 (46.7%)	39 (53.4%)
Completely	52 (19.9%)	3 (14.3%)	40 (24.0%)	9 (12.3%)
**Perception that work is meaningful**				
Disagree/ Neutral	19 (7.3%)	0 (0.0%)	13 (7.8%)	6 (8.2%)
Agree	138 (52.9%)	13 (61.9%)	91 (54.5%)	34 (46.6%)
Strongly Agree	104 (39.8%)	8 (38.1%)	63 (37.7%)	33 (45.2%)
**Highest educational level**				
Pre-University & Vocational Education	19 (7.3%)	0 (0.0%)	6 (3.6%)	13 (17.8%)
Polytechnic/ Diploma/ Equivalent	65 (24.9%)	0 (0.0%)	58 (34.7%)	7 (9.6%)
University Degree	141 (54.0%)	14 (66.7%)	89 (53.3%)	38 (52.1%)
Postgraduate Degree	36 (13.8%)	7 (33.3%)	14 (8.4%)	15 (20.5%)
**Level of burnout** ^**b**^				
1	41 (15.7%)	5 (23.8%)	28 (16.8%)	8 (11.0%)
2	145 (55.6%)	11 (52.4%)	94 (56.3%)	40 (54.8%)
3	56 (21.5%)	4 (19.0%)	36 (21.6%)	16 (21.9%)
4	11 (4.2%)	1 (4.8%)	7 (4.2%)	3 (4.1%)
5	8 (3.1%)	0 (0.0%)	2 (1.2%)	6 (8.2%)

^a^Junior Nursing staff refer to nursing aides, enrolled nurses and senior enrolled nurses. Senior Nursing staff refer to staff nurses, senior staff nurses, advanced practitioner nurses and nursing managers

^b^Level of burnout was assessed using the Single Item Burnout Measure, with responses on an ordinal scale. (see Additional File 1, Supplementary Section S6 for details)

Data are reported as count (%) unless otherwise stated

### Psychometric performance of the PPOS scale

Internal consistency for the overall PPOS was acceptable in our sample (Cronbach’s *α* = 0.794). However, the subscales demonstrated lower reliability (‘Caring’ domain *α* = 0.613; ‘Sharing’ domain *α* = 0.704). Item-level analysis revealed variability in performance. Whilst several items showed moderate to strong item-total correlation (> 0.3), three reverse-coded items (Q9, Q13 and Q17) demonstrated near-zero or negative item-total correlations (see Supplementary Table S1.2). Exploratory Factor Analysis (EFA) further indicated a relatively weak factor structure in this cohort. A three-factor solution explained a cumulative variance of only 31.8%, with the primary factor accounting for 18.36% of the variance. Furthermore, many items, particularly the reverse-coded ones, had high uniqueness (> 0.7), suggesting that a significant proportion of the variance in responses was not captured by the underlying factors. Given these psychometric properties and the variability in reproducing the original two-factor structure in international settings [53], the PPOS was treated as a unidimensional composite measure for the purpose of this analysis, providing a global index of patient-centred orientation. Detailed psychometric results are in Additional File 1, Supplementary Section S1 and S2.

### Descriptive analysis of PPOS scores across professional groups

The overall mean PPOS score among participants was 3.75 points (*SD* = 0.63). Physicians had the highest mean score (*M* = 4.29, *SD* = 0.49), followed by therapists (*M* = 4.12, *SD* = 0.46), senior nursing staff (*M* = 3.73, *SD* = 0.63), therapy aides (*M* = 3.69, *SD* = 0.48), and junior nursing staff (*M* = 3.36, *SD* = 0.54) (Table 2). There were no missing data.

**Table 2:** Mean PPOS scores for the total population and by professional roles

Group	*n*	PPOS	
		Mean	SD
Overall	261	**3.75**	0.63
Physician	21	4.29	0.49
Therapist	45	4.12	0.46
Senior Nursing Staff^a^	103	3.73	0.63
Therapy Aide	28	3.69	0.48
Junior Nursing Staff^a^	64	3.36	0.54

^a^Junior Nursing staff refer to nursing aides, enrolled nurses and senior enrolled nurses. Senior Nursing staff refer to staff nurses, senior staff nurses, advanced practitioner nurses and nursing managers

### Multivariable linear regression analysis

Comparing the initial demographic-adjusted single-variable models (Supplementary Tables S4.1 - S4.18) to the final multivariable analysis (Table 3) clarified the relative influence of the candidate predictors. While factors such as postgraduate education, work meaningfulness, and daily patient volume were significant in the initial demographic-adjusted single-variable models, these associations did not retain significance in the final analysis. In contrast, a hospital culture that promotes PCC, professional role, and burnout levels remained significant throughout all modelling stages (see Additional File 1, Supplementary Section S4 and S5).

**Table 3:** Multivariable linear regression analysis of factors associated with PPOS scores

				95% confidence interval		
	Unstandardised Estimate		SE	Lower	Upper	*p*-value
**Age category (ref: 21–29 years)**						
30–39 years	-0.01		0.10	-0.20	0.18	0.904
40–49 years	0.10		0.11	-0.12	0.31	0.373
≥ 50 years	-0.10		0.13	-0.35	0.15	0.418
**Gender (ref: Female)**						
Male	-0.06		0.10	-0.25	0.13	0.532
**Educational level (ref: Pre-U and vocational education)**						
Polytechnic/ Diploma	0.22		0.17	-0.12	0.56	0.212
University Degree	0.20		0.17	-0.14	0.53	0.242
Postgraduate Degree	0.36		0.19	-0.02	0.74	0.064
**Perception that the hospital culture promotes PCC (ref: Not at all/To a small extent)**						
To some extent	0.26		0.17	-0.06	0.59	0.113
To a great extent	0.50		0.16	0.18	0.82	**0.002**
Completely	0.61		0.17	0.27	0.95	**< 0.001**
**Professional role (ref: Physician)**						
Junior Nursing Staff^a^	-0.79		0.15	-1.09	-0.49	**< 0.001**
Senior Nursing Staff^a^	-0.55		0.15	-0.84	-0.26	**< 0.001**
Therapist	-0.10		0.16	-0.40	0.21	0.542
Therapy Aide	-0.39		0.19	-0.76	-0.02	**0.039**
**Most common type of patients served (ref: Palliative patients)**						
Dementia patients	-0.11		0.12	-0.35	0.13	0.369
Rehabilitation patients	-0.11		0.10	-0.31	0.08	0.257
Wound care patients	-0.23		0.12	-0.48	0.00	0.050
Others	-0.01		0.21	-0.42	0.40	0.957
**Perception that work is meaningful (ref: Disagree/ Neutral)**						
Agree	0.07		0.13	-0.20	0.33	0.612
Strongly Agree	0.22		0.14	-0.05	0.49	0.108
**Level of burnout**^**b**^ **(ref: 1)**						
2	0.26		0.10	0.06	0.47	**0.012**
3	0.12		0.12	-0.12	0.36	0.316
4	0.06		0.19	-0.33	0.43	0.793
5	0.07		0.22	-0.36	0.49	0.768
**Number of patients seen per day**	0.00		0.01	-0.01	0.01	0.697

^a^Junior Nursing staff refer to nursing aides, enrolled nurses and senior enrolled nurses. Senior Nursing staff refer to staff nurses, senior staff nurses, advanced practitioner nurses and nursing managers

^b^Level of burnout was assessed using the Single Item Burnout Measure, with responses on an ordinal scale

Perceptions of hospital culture as promoting PCC were significantly associated with patient-centred orientation. Respondents who perceived that their hospital promotes PCC ‘to a great extent’ or ‘completely’ scored higher on the PPOS than those who reported ‘to a small extent’ or ‘not at all’.

Professional role was also a strong predictor of PPOS scores. Compared to physicians (the reference group), junior nurses scored 0.79 points lower (*SE* = 0.15, *p* < 0.001), senior nurses scored 0.55 points lower (*SE* = 0.15, *p* < 0.001), and therapy aides scored 0.39 points lower (*SE* = 0.19, *p* = 0.039).

An unexpected association was observed between burnout level and patient-centred orientation. Participants who reported occasional stress without burnout (Category 2 of the burnout scale) scored 0.26 points higher on the PPOS than those reporting no symptoms of burnout (*SE* = 0.10, *p* = 0.012).

The final multivariable linear regression model demonstrated good overall fit (*F* = 5.49, *p* < 0.001) explaining 30.2% of the variance in PPOS scores (Adjusted *R²* = 0.302) (see Additional File 1, Supplementary Section S5).

## Discussion

### Principal findings

This study evaluated patient-centred orientation among multidisciplinary healthcare professionals in a Singaporean community hospital. Using the Patient-Practitioner Orientation Scale (PPOS), healthcare professionals demonstrated a mean orientation score of 3.75 points, which falls within the mid-range of scores reported in international literature [20, 28–30, 33, 36, 54–56]. Multivariable analysis identified professional role, a hospital culture that promotes PCC, and occasional stress without burnout as factors independently associated with patient-centred orientation.

### Psychometric considerations

The difficulty in reproducing a robust factor structure for the PPOS has also been reported in a recent scoping review of translated PPOS versions [53]. Alongside sub-optimal internal consistency of the ‘Caring’ subscale, this suggests that the traditional ‘Sharing’ and ‘Caring’ domains of the original scale may not be strictly applicable within our Singaporean community hospital cohort. This likely reflects a conceptual overlap influenced by professional hierarchy and cultural diversity. In our setting, junior nurses and therapy aides primarily execute care plans rather than lead decision-making; consequently, survey items focusing on the ‘sharing’ of power may be perceived as outside their job scope. This complexity is further compounded by a multinational workforce trained in diverse regions, including Myanmar, the Philippines, Malaysia, and China. Such diversity can lead to differing cultural conceptualisations of the healthcare professional’s role and what constitutes PCC, often rooted in the clinical traditions of their respective home countries [57, 58]. Furthermore, varying levels of English proficiency among non-native speakers may have influenced the interpretation of specific items.

### Comparison with existing literature

#### Comparison of mean PPOS scores

The mean PPOS score in our study (3.75 points) lies within the mid-range of international findings. Higher scores (4.02–4.8 points) have been reported in studies from North America, Asia (i.e., Singapore, Malaysia, Saudi Arabia), and Europe [20, 28, 29, 33, 36, 54], while lower scores (< 3.75 points) have been observed in other parts of Asia (i.e., Kazakhstan, Iran, Korea) [30, 55, 56], reflecting possible cultural and systemic influences. For instance, hierarchical and paternalistic healthcare cultures, institutional emphasis on PCC, and structural constraints such as workload or time pressure may contribute to these differences [59–61]. Nonetheless, our findings indicate room for improvement, particularly as Singapore’s health system moves towards PCC.

### Factors associated with higher or lower PPOS scores

#### Professional role

Differences in PPOS scores by role were the most pronounced and remained significant in multivariable analysis. Physicians scored higher than nurses and therapy aides, a pattern observed in some international studies [33, 36]. This disparity may reflect differences in clinical hierarchy, decision-making authority, and perceived empowerment across professional groups. For instance, in Singapore, physicians lead shared decision-making, whereas nurses and therapy aides often operate within protocol-driven or task-oriented scopes of practice. Enhancing professional empowerment, through giving therapy aides and nurses more liberty and ownership to integrate elements of shared decision-making and empathetic communication strategies into their daily practice, may foster more participation in patient engagement. Several studies suggest that training can also improve patient-centred orientation [54, 62]. Strengthening curricula and professional development for nurses and therapy aides could thus help bridge this gap in orientation and promote a more consistent patient-centred orientation across the multidisciplinary team.

#### Hospital culture supportive of PCC

Participants who perceived that their hospital culture promotes PCC ‘to a great extent’ or ‘completely’ had significantly higher PPOS scores. This finding is consistent with previous studies showing that organisational cultures characterised by openness, trust, interprofessional collaboration, and leadership commitment to PCC, have been associated with improved care delivery [63, 64]. When healthcare professionals perceive genuine institutional support, they are more likely to embody and advocate for patient-centred values in their everyday practice [65].

### Burnout

A significant association was found between participants reporting symptoms of occasional stress without burnout (Category 2 of the burnout scale) and higher PPOS scores. This finding appears counterintuitive as physician burnout is typically linked to poorer quality of care and less favourable patient-reported experiences [50, 51].

Nonetheless, this association should be interpreted cautiously. Because this study was cross-sectional, it precludes causal or temporal inference. It is therefore equally plausible that healthcare professionals with stronger patient-centred orientation may be more emotionally invested in patient care and consequently report greater work-related stress, or that participants in Category 2 represent healthcare professionals experiencing manageable levels of work-related stress before the reductions in empathy that have been reported in persistent burnout [66]. Residual confounding from unmeasured individual characteristics, such as personality traits or coping strategies, may also contribute to the observed association. Longitudinal studies are needed to clarify these relationships.

### Strengths and limitations

This study has several strengths. It is the first in Singapore to use the PPOS in a community hospital, and one with a unique ecosystem spanning inpatient to outpatient and home-care services. The multidisciplinary sample of physicians, nurses, and therapists also enabled comparisons across roles. Additionally, inclusion of psychosocial variables such as burnout, institutional culture, and workload allowed us to identify potential contributory factors.

Several limitations warrant consideration. As a single-centre study, the findings may not be fully generalisable to other healthcare settings, and the cross-sectional design precludes causal inference. Despite a 79.1% response rate, non-response bias remains possible, as non-participants may hold less patient-centred orientation. Social desirability bias is also an inherent limitation of self-reported surveys like the PPOS, where responses may reflect perceived professional ideals rather than actual orientation. We attempted to minimize these risks through voluntary participation and guaranteed anonymity.

Additionally, as the PPOS assesses self-reported orientation rather than observed practice, it remains unclear whether higher scores translate into actual patient-centred behaviours. In fact, qualitative evaluations on the practice and translation of PCC consistently demonstrate that multilevel barriers can result in an attitude–behaviour gap [38, 67]. Furthermore, patient-centred orientation is a complex, multidimensional construct that extends beyond the ‘Sharing’ and ‘Caring’ domains captured by the PPOS [3]. The relatively unstable factor structure observed in this sample also suggests that contextual drivers of patient-centred orientation in this population may not be fully captured by the scale.

There was also a notable imbalance in group sizes across professional roles, with substantially fewer physicians (*n* = 21) compared to other roles. While this reflects local staffing ratios within our community hospital, the small physician sample reduced the precision of estimates for comparisons involving physicians, who served as the reference group in the regression analysis. Consequently, coefficient estimates may have been more sensitive to individual outliers and may have had insufficient statistical power to detect small or moderate difference between physicians and other professional groups. These findings should therefore be interpreted with appropriate caution and validated in larger, more balanced cohorts. The inclusion of a large number of predictors relative to the sample size (*n* = 261) also increased the risk of model overfitting and unstable coefficient estimates. Although a more parsimonious model was used, this may have excluded variables that are theoretically meaningful despite not reaching statistical significance in this dataset.

### Implications for research and practice

Whilst bearing in mind the above limitations, our findings nonetheless have important implications for clinical practice, workforce development, and leadership in community and rehabilitative care settings.

First, targeted strategies are needed to enhance patient-centred orientation among nurses and therapy aides. Training in elements such as communication, empathy, psychosocial care, and shared decision-making may help bridge this gap.

Second, fostering an institutional culture that promotes PCC is important. Leadership should model PCC values, reinforce them through team communications, and recognise relational aspects of care. Embedding PCC competencies into performance appraisals and professional development frameworks can help align organisational values with daily practice [65].

Third, sustained attention to staff well-being is also crucial. Burnout, though multifactorial, may undermine PCC. While mild stress was associated with higher patient-centred orientation in this study, established burnout has been associated with poorer patient-centred care in previous research [50, 51, 66]. Promoting manageable workloads, peer support, and psychological safety therefore remain important considerations.

At a policy level, these findings resonate with Singapore’s Healthier SG reforms, which are grounded in PCC principles. Embedding PCC training, leadership accountability, and staff wellness into institutional structures will be essential to achieving these goals across the care continuum.

### Future research

Further research should investigate how patient-centred orientation translates into clinical behaviours. Specifically, mixed methods approaches that combine PPOS scores with qualitative interviews, direct clinical observations, or patient-reported experience and outcome measures (PREMs and PROMs) would clarify whether care orientation translates into tangible practice behaviours. Regarding instrument development, there is a need to culturally adapt the PPOS or develop tools tailored to multicultural Asian settings so as to accurately capture the dimensions of patient-centred orientation in these contexts. Finally, longitudinal, multi-site studies with larger cohorts are needed to validate the findings identified in this study.

## Conclusion

This study provides one of the first insights into patient-centred orientation across multiple professional groups in a Singapore community hospital. Professional role, a hospital culture that promotes PCC, and occasional stress were independently associated with patient-centred orientation. Targeted training for nurses and therapy aides, stronger organisational commitment to PCC, and proactive strategies to support staff well-being may represent promising areas for future interventions to strengthen patient-centred orientation. The reverse, namely that the direct promotion of patient-centeredness may improve professional wellbeing among healthcare staff on multiple levels may also be considered based on these cross-sectional findings. Further studies are needed to explore these avenues and establish whether self-reported patient-centred orientation also translates into measurable patient-centred practices. By contributing to the limited local evidence base, this study highlights priorities for future research and organisational initiatives to strengthen PCC across the care continuum in Singapore.

## Supplementary Information

Below is the link to the electronic supplementary material.

Supplementary Material 1

## Data Availability

All aggregate data supporting the findings of this article are included within the article and its additional file. Raw data are available from the corresponding author upon reasonable request.

## Abbreviations

PCC: Patient-Centred Care
PPOS: Patient-Practitioner Orientation Scale
PREMs: Patient-Reported Experience Measures
PROMs: Patient-Reported Outcome Measures
SLH: St Luke’s Hospital
