# Supplementary material for: Patient-centred orientation among healthcare professionals in a Singapore community hospital
Source: BMC Health Serv Res. 2026 Jul 27;26:1557. doi: 10.1186/s12913-026-15252-0 (PMC13746713; doi:10.1186/s12913-026-15252-0)
Supplement: Supplementary file 1 [file 12913_2026_15252_MOESM1_ESM.docx]

SUPPLEMENTARY MATERIAL

CONTENTS

[Supplementary Section S1 – Reliability Statistics	2](#_Toc232194347)

[Supplementary Section S2 – Exploratory Factor Analysis (EFA) Results	3](#_Toc232194348)

[Supplementary Section S3: Multiple Linear Regression: Model Selection	5](#_Toc232194349)

[Supplementary Section S4: Demographic-Adjusted Single-Variable Models	7](#_Toc232194350)

[Supplementary Section S5: Multiple Linear Regression: Model Comparisons and Model 2	25](#_Toc232194351)

[Supplementary Section S6: Questionnaire and patient information sheet and consent form	29](#_Toc232194352)

## Supplementary Section S1 – Reliability Statistics

**Supplementary Table S1.1:** Overall Cronbach’s Alpha for PPOS Scale

| **Scale** | **Cronbach's α** |
| --- | --- |
| PPOS (Total Scale) | 0.794 |

**Supplementary Table S1.2:** Cronbach’s Alpha - Item-Level Analysis

| **Item** | **Mean** | **SD** | **Item-Total Correlation** | **Cronbach's α if Item Deleted** |
| --- | --- | --- | --- | --- |
| Q1 PPOS | 3.28 | 1.35 | 0.50 | 0.78 |
| Q2 PPOS | 2.93 | 1.04 | 0.32 | 0.79 |
| Q3 PPOS | 2.21 | 1.16 | 0.19 | 0.79 |
| Q4 PPOS | 4.43 | 1.47 | 0.58 | 0.77 |
| Q5 PPOS | 3.98 | 1.56 | 0.49 | 0.78 |
| Q6 PPOS | 4.22 | 1.46 | 0.57 | 0.77 |
| Q7 PPOS | 4.75 | 1.39 | 0.60 | 0.77 |
| Q8 PPOS | 3.30 | 1.28 | 0.49 | 0.78 |
| Q9 PPOS-R | 4.34 | 1.38 | -0.01 | 0.81 |
| Q10 PPOS | 2.70 | 1.15 | 0.33 | 0.79 |
| Q11 PPOS | 4.11 | 1.39 | 0.48 | 0.78 |
| Q12 PPOS | 3.76 | 1.33 | 0.41 | 0.78 |
| Q13 PPOS-R | 4.55 | 1.14 | 0.01 | 0.80 |
| Q14 PPOS | 3.67 | 1.44 | 0.45 | 0.78 |
| Q15 PPOS | 3.46 | 1.39 | 0.57 | 0.77 |
| Q16 PPOS | 4.64 | 1.50 | 0.48 | 0.78 |
| Q17 PPOS-R | 4.30 | 1.20 | -0.11 | 0.81 |
| Q18 PPOS | 2.86 | 1.19 | 0.25 | 0.79 |

## Supplementary Section S2 – Exploratory Factor Analysis (EFA) Results

**Supplementary Table S2.1** Factor Loadings (Maximum Likelihood Extraction with Oblimin Rotation)

| **Item** | **Factor 1** | **Factor 2** | **Uniqueness** |
| --- | --- | --- | --- |
| Q1 PPOS | 0.39 |  | 0.69 |
| Q2 PPOS |  | 0.37 | 0.78 |
| Q3 PPOS |  | 0.61 | 0.61 |
| Q4 PPOS | 0.74 |  | 0.49 |
| Q5 PPOS | 0.52 |  | 0.68 |
| Q6 PPOS | 0.66 |  | 0.55 |
| Q7 PPOS | 0.72 |  | 0.48 |
| Q8 PPOS | 0.38 |  | 0.70 |
| Q9 PPOS-R |  |  | 0.93 |
| Q10 PPOS |  | 0.37 | 0.74 |
| Q11 PPOS | 0.52 |  | 0.63 |
| Q12 PPOS |  | 0.55 | 0.58 |
| Q13 PPOS-R |  |  | 0.90 |
| Q14 PPOS | 0.36 |  | 0.71 |
| Q15 PPOS | 0.38 | 0.35 | 0.56 |
| Q16 PPOS | 0.49 |  | 0.66 |
| Q17 PPOS-R |  | -0.51 | 0.73 |
| Q18 PPOS |  |  | 0.86 |

**Supplementary Table S2.2** Factor Statistics

| **Factor** | **SS Loadings** | **% of Variance** | **Cumulative %** |
| --- | --- | --- | --- |
| 1 | 3.31 | 18.36 | 18.40 |
| 2 | 1.44 | 7.98 | 26.30 |
| 3 | 0.98 | 5.43 | 31.80 |

## Supplementary Section S3: Multiple Linear Regression: Model Selection

Multiple linear regression (MLR) was used to identify independent predictors of PPOS scores. Three models were developed:

1. **Demographic-Adjusted Single-Variable Models:** wherein each predictor was individually adjusted for age, gender, and education to screen for potential confounders
2. **Full model (Model 2):** wherein all variables were included
3. **Final model (Model 3):** a more parsimonious model developed by only including variables that remained statistically significant (p <0.05) in the demographic-adjusted single-variable models after adjustment for age, gender and education

Model Comparison and Selection

"The variable selection process aimed to balance the inclusion of theoretically relevant factors with the need to maintain model stability given the sample size. Twenty variables (represented by 54 predictors after dummy coding) were identified *a priori* based on clinical plausibility and prior literature.

Given the relatively large number of predictors in relation to the sample size (N = 261), a staged modelling approach was used to reduce the risk of overfitting and unstable coefficient estimates. In the first step (**Demographic-Adjusted Single-Variable Models**), each candidate predictor was individually adjusted for core demographic covariates (age, gender, and education) to identify variables showing independent associations with PPOS scores. In the second step (**Model 2**), following the enter method, all predictors were entered simultaneously into a full exploratory model to assess the overall variance explained and examine the combined behavior of all candidate variables.

Because the full model contained a large number of predictors relative to the sample size, manual backward elimination was performed to derive a more parsimonious model (**Model 3**) to improve model stability, reduce unnecessary model complexity, and facilitate interpretation. Model 3 included variables that remained significant after adjustment in the demographic-adjusted single-variable models.

Model 2, which included all variables (n=54 predictors), explained slightly more variance (R²=0.479) than Model 3 (R^2^=0.369) but had a comparable adjusted R² (0.314 for Model 2 vs 0.302 for Model 3). While Model 2 had a lower Akaike Information Criterion than Model 3 (AIC = 396 for Model 2 vs 430 for Model 3), it had a substantially higher Bayesian Information Criterion (BIC = 588 for Model 2 vs 526 for Model 3), indicating greater model complexity. Model 3 retained only 25 predictors and demonstrated improved parsimony. Model 3 also achieved a higher F-statistic than Model 2 (5.49 vs. 2.90). Taken together these model characteristics, suggested better model efficiency and interpretability of Model 3, which was selected as the final model for interpretation. See **Supplementary Section S5** for more details.

Model assumptions were checked and adequately met. Multicollinearity was assessed via Variance Inflation Factor (VIF), with all VIF values < 2. Residuals were assessed for normality (via Q–Q plots and Shapiro–Wilk test), linearity, and homoscedasticity. The Durbin–Watson statistic confirmed independence of errors, and Cook’s distance values were all < 0.1, indicating no influential observations. All analyses were performed using Jamovi Version 2.4. A two-tailed p-value of < 0.05 was considered statistically significant.

## Supplementary Section S4: Demographic-Adjusted Single-Variable Models

**Note for all tables in this section:** “ref” indicates the reference category for each categorical predictor. P-values < 0.05 were considered statistically significant.

**Supplementary Table S4.1** Association between perceived hospital culture promoting patient-centred care (PCC) and PPOS scores, adjusted for age, gender, and highest educational level.

| **Predictor** | | | **Unstandardised Estimate** | **Standard Error** | ***t* statistic** | ***p*-value** |
| --- | --- | --- | --- | --- | --- | --- |
|  |  |  |  |  |  |  |
| **Intercept** | |  | 3.07 | 0.21 | 14.41 | < 0.001 |
| **Age (ref: 21 - 29 years)** | | | | | | |
|  | | 30 - 39 years | -0.05 | 0.10 | -0.48 | 0.63 |
|  | | 40 - 49 years | 0.10 | 0.11 | 0.89 | 0.377 |
|  | | ≥ 50 years | -0.06 | 0.13 | -0.47 | 0.64 |
| **Gender (ref: female)** | | | | | | |
|  | | male | 0.02 | 0.10 | 0.25 | 0.805 |
| **Highest Educational Level  (ref: Pre-U and Vocational Education)** | | | | | | |
|  | Polytechnic/Diploma/ Equivalent | | 0.09 | 0.16 | 0.56 | 0.575 |
|  | University Degree | | 0.28 | 0.16 | 1.77 | 0.078 |
|  | Postgraduate Degree | | 0.62 | 0.18 | 3.50 | < 0.001 |
| **Hospital Culture Promotes Patient Centred Care  (ref: Not at all / To a small extent)** | | | | | | |
|  | | To some extent | 0.22 | 0.18 | 1.23 | 0.22 |
|  | | To a great extent | 0.53 | 0.17 | 3.10 | 0.002 |
|  | | Completely | 0.51 | 0.18 | 2.79 | 0.006 |

**Supplementary Table S4.2** Association between burnout level and PPOS scores, adjusted for age, gender, and education.

| **Predictor** | | **Unstandardised Estimate** | **Standard Error** | ***t* statistic** | ***p*-value** |
| --- | --- | --- | --- | --- | --- |
|  |  |  |  |  |  |
| **Intercept** |  | 3.18 | 0.18 | 17.33 | < 0.001 |
| **Age (ref: 21 - 29 years)** | | | | | |
|  | 30 - 39 years | -0.10 | 0.10 | -1.03 | 0.306 |
|  | 40 - 49 years | 0.04 | 0.12 | 0.31 | 0.759 |
|  | ≥ 50 years | -0.01 | 0.13 | -0.08 | 0.939 |
| **Gender (ref: female)** | |  | | | |
|  | male | 0.05 | 0.10 | 0.52 | 0.604 |
| **Highest Educational Level (ref: Pre-U and Vocational Education)** | |  | | | |
|  | Polytechnic/Diploma/ Equivalent | 0.22 | 0.17 | 1.30 | 0.194 |
|  | University Degree | 0.39 | 0.16 | 2.45 | 0.015 |
|  | Postgraduate Degree | 0.72 | 0.18 | 4.02 | < 0.001 |
| **Level of burnout^#^ (ref: 1)** | |  | | | |
|  | 2 | 0.30 | 0.11 | 2.71 | 0.007 |
|  | 3 | 0.25 | 0.13 | 1.96 | 0.052 |
|  | 4 | 0.05 | 0.21 | 0.24 | 0.811 |
|  | 5 | 0.26 | 0.23 | 1.12 | 0.264 |

*Note # Level of burnout was assessed using the Single Item Burnout Measure, with responses on an ordinal scale: 1= I enjoy my work. I have no symptoms of burnout; 2= Occasionally I am under stress, and I don’t always have as much energy as I once did, but I don’t feel burned out; 3= I am definitely burning out and have one or more symptoms of burnout, such as physical and emotional exhaustion; 4=The symptoms of burnout that I’m experiencing won’t go away. I think about frustration at work a lot; 5= I feel completely burned out and often wonder if I can go on. I am at the point where I may need some changes or may need to seek some sort of help.*

**Supplementary Table S4.3** Association between the perception that workload is manageable and PPOS scores, adjusted for age, gender, and education.

| **Predictor** | | **Unstandardised Estimate** | **Standard Error** | ***t* statistic** | ***p*-value** |
| --- | --- | --- | --- | --- | --- |
|  |  |  |  |  |  |
| **Intercept** |  | 3.74 | 0.30 | 12.56 | < 0.001 |
| **Age (ref: 21 - 29 years)** | |  | | | |
|  | 30 - 39 years | -0.02 | 0.10 | -0.23 | 0.821 |
|  | 40 - 49 years | 0.10 | 0.11 | 0.90 | 0.369 |
|  | ≥ 50 years | 0.01 | 0.13 | 0.11 | 0.911 |
| **Gender (ref: female)** | |  |  |  |  |
|  | male | 0.01 | 0.10 | 0.08 | 0.936 |
| **Highest Educational Level  (ref: Pre-U and Vocational Education)** | | | | | |
|  | Polytechnic/Diploma/ Equivalent | 0.21 | 0.17 | 1.26 | 0.208 |
|  | University Degree | 0.40 | 0.16 | 2.49 | 0.014 |
|  | Postgraduate Degree | 0.75 | 0.18 | 4.16 | < 0.001 |
| **Workload is manageable  (ref: Strongly Disagree)** | | | | | |
|  | Disagree | -0.29 | 0.28 | -1.05 | 0.295 |
|  | Neutral | -0.43 | 0.26 | -1.65 | 0.1 |
|  | Agree | -0.41 | 0.25 | -1.64 | 0.102 |
|  | Strongly agree | -0.25 | 0.28 | -0.90 | 0.37 |

**Supplementary Table S4.4** Association between the perception that work is satisfying and PPOS scores, adjusted for age, gender, and education.

| **Predictor** | | **Unstandardised Estimate** | **Standard Error** | ***t* statistic** | ***p*-value** |
| --- | --- | --- | --- | --- | --- |
|  |  |  |  |  |  |
| **Intercept** |  | 3.06 | 0.32 | 9.68 | <0.001 |
| **Age (ref: 21 - 29 years)** | |  | | | |
|  | 30 - 39 years | -0.03 | 0.10 | -0.25 | 0.8 |
|  | 40 - 49 years | 0.08 | 0.11 | 0.69 | 0.49 |
|  | ≥ 50 years | -0.02 | 0.13 | -0.15 | 0.884 |
| **Gender (ref: female)** | |  |  |  |  |
|  | male | 0.00 | 0.10 | 0.02 | 0.982 |
| **Highest Educational Level  (ref: Pre-U and Vocational Education)** | | | | | |
|  | Polytechnic/Diploma/Equivalent | 0.20 | 0.17 | 1.19 | 0.237 |
|  | University Degree | 0.41 | 0.16 | 2.61 | 0.01 |
|  | Postgraduate Degree | 0.77 | 0.18 | 4.31 | < 0.001 |
| **Work is satisfying (ref: Strongly Disagree/ Disagree)** | | | | | |
|  | Neutral | 0.12 | 0.29 | 0.42 | 0.678 |
|  | Agree | 0.35 | 0.28 | 1.27 | 0.204 |
|  | Strongly agree | 0.34 | 0.29 | 1.19 | 0.235 |

**Supplementary Table S4.5** Association between the perception that work is meaningful and PPOS scores, adjusted for age, gender, and education.

| **Predictor** | | **Unstandardised Estimate** | **Standard Error** | ***t* statistic** | ***p*-value** |
| --- | --- | --- | --- | --- | --- |
|  |  |  |  |  |  |
| **Intercept** |  | 3.16 | 0.21 | 15.05 | <0.001 |
| **Age (ref: 21 - 29 years)** | |  | | | |
|  | 30 - 39 years | -0.06 | 0.10 | -0.65 | 0.515 |
|  | 40 - 49 years | 0.05 | 0.11 | 0.49 | 0.627 |
|  | ≥ 50 years | -0.04 | 0.13 | -0.32 | 0.75 |
| **Gender (ref: female)** | |  |  |  |  |
|  | male | -0.03 | 0.10 | -0.34 | 0.735 |
| **Highest Educational Level  (ref: Pre-U and Vocational Education)** | | | | | |
|  | Polytechnic/Diploma/ Equivalent | 0.18 | 0.16 | 1.11 | 0.269 |
|  | University Degree | 0.41 | 0.15 | 2.65 | 0.009 |
|  | Postgraduate Degree | 0.75 | 0.17 | 4.28 | < 0.001 |
| **Work is meaningful  (ref: Disagree/ Neutral)** | | | | | |
|  | Agree | 0.17 | 0.14 | 1.15 | 0.253 |
|  | Strongly agree | 0.41 | 0.15 | 2.76 | 0.006 |

**Supplementary Table S4.6** Association between the perception of support at work for difficult patient interactions and PPOS scores, adjusted for age, gender, and education.

| **Predictor** | | **Unstandardised Estimate** | **Standard Error** | ***t* statistic** | ***p*-value** |  |
| --- | --- | --- | --- | --- | --- | --- |
|  |  |  |  |  |  |  |
| **Intercept** |  | 3.32 | 0.22 | 15.30 | < 0.001 |  |
| **Age (ref: 21 - 29 years)** | |  | | | |  |
|  | 30 - 39 years | -0.05 | 0.10 | -0.52 | 0.605 |  |
|  | 40 - 49 years | 0.07 | 0.11 | 0.63 | 0.531 |  |
|  | ≥ 50 years | -0.04 | 0.14 | -0.32 | 0.752 |  |
| **Gender (ref: female)** | |  |  |  |  |  |
|  | male | 0.02 | 0.10 | 0.19 | 0.851 |  |
| **Highest Educational Level  (ref: Pre-U and Vocational Education)** | | | | | |  |
|  | Polytechnic/Diploma/ Equivalent | 0.13 | 0.17 | 0.78 | 0.439 |  |
|  | University Degree | 0.35 | 0.16 | 2.24 | 0.026 |  |
|  | Postgraduate Degree | 0.70 | 0.18 | 3.91 | < 0.001 |  |
| **Support at work for difficult patient interactions  (ref: Not at all / To a small extent)** | | | | | |  |
|  | To some extent | 0.04 | 0.17 | 0.24 | 0.808 |  |
|  | To a great extent | 0.22 | 0.17 | 1.33 | 0.185 |  |
|  | Completely | 0.07 | 0.18 | 0.40 | 0.693 |  |

**Supplementary Table S4.7** Association between the frequency of facing language barriers during patient interactions and PPOS scores, adjusted for age, gender, and education.

| **Predictor** | | **Unstandardised Estimate** | **Standard Error** | ***t* statistic** | ***p*-value** |
| --- | --- | --- | --- | --- | --- |
|  |  |  |  |  |  |
| **Intercept** |  | 3.48 | 0.27 | 12.77 | < 0.001 |
| **Age (ref: 21 - 29 years)** | |  | | | |
|  | 30 - 39 years | -0.06 | 0.10 | -0.63 | 0.529 |
|  | 40 - 49 years | 0.06 | 0.12 | 0.56 | 0.576 |
|  | ≥ 50 years | -0.02 | 0.14 | -0.15 | 0.878 |
| **Gender (ref: female)** | |  |  |  |  |
|  | male | 0.00 | 0.10 | 0.02 | 0.981 |
| **Highest Educational Level  (ref: Pre-U and Vocational Education)** | | | | | |
|  | Polytechnic/Diploma/ Equivalent | 0.19 | 0.17 | 1.11 | 0.268 |
|  | University Degree | 0.39 | 0.17 | 2.34 | 0.02 |
|  | Postgraduate Degree | 0.75 | 0.19 | 4.03 | < 0.001 |
| **Frequency of language barriers  during patient interactions (ref: Not at all)** | | | | | |
|  | Rarely | -0.02 | 0.29 | -0.06 | 0.95 |
|  | Sometimes | -0.13 | 0.27 | -0.47 | 0.642 |
|  | Frequently | 0.02 | 0.29 | 0.07 | 0.944 |
|  | Always | 0.16 | 0.39 | 0.41 | 0.685 |

**Supplementary Table S4.8** Association between the frequency of facing cultural barriers during patient interactions and PPOS scores, adjusted for age, gender, and education.

| **Predictor** | | **Unstandardised Estimate** | **Standard Error** | ***t* statistic** | ***p*-value** |
| --- | --- | --- | --- | --- | --- |
|  |  |  |  |  |  |
| **Intercept** |  | 3.43 | 0.21 | 16.24 | < 0.001 |
| **Age (ref: 21 - 29 years)** | |  | | | |
|  | 30 - 39 years | -0.04 | 0.10 | -0.37 | 0.712 |
|  | 40 - 49 years | 0.09 | 0.11 | 0.76 | 0.446 |
|  | ≥ 50 years | 0.00 | 0.13 | 0.01 | 0.989 |
| **Gender (ref: female)** | |  |  |  |  |
|  | male | 0.00 | 0.10 | 0.02 | 0.981 |
| **Highest Educational Level  (ref: Pre-U and Vocational Education)** | | | | | |
|  | Polytechnic/Diploma/ Equivalent | 0.18 | 0.17 | 1.12 | 0.265 |
|  | University Degree | 0.39 | 0.16 | 2.48 | 0.014 |
|  | Postgraduate Degree | 0.74 | 0.18 | 4.13 | < 0.001 |
| **Frequency of cultural barriers during  patient interactions (ref: Not at all)** | | | | | |
|  | Rarely | 0.02 | 0.17 | 0.12 | 0.907 |
|  | Sometimes | -0.09 | 0.16 | -0.57 | 0.57 |
|  | Always | 0.25 | 0.23 | 1.09 | 0.275 |

**Supplementary Table S4.9** Association between the level of preoccupations and troubles in one’s private life and PPOS scores, adjusted for age, gender, and education.

| **Predictor** | | **Unstandardised Estimate** | **Standard Error** | ***t* statistic** | | ***p*-value** |  |
| --- | --- | --- | --- | --- | --- | --- | --- |
|  |  |  |  |  |  |  |  |
| **Intercept** |  | 3.30 | 0.20 | | 16.25 | < 0.001 |  |
| **Age (ref: 21 - 29 years)** | |  | | | | |  |
|  | 30 - 39 years | -0.02 | 0.11 | | -0.22 | 0.823 |  |
|  | 40 - 49 years | 0.10 | 0.12 | | 0.86 | 0.39 |  |
|  | ≥ 50 years | 0.03 | 0.14 | | 0.23 | 0.82 |  |
| **Gender (ref: female)** | |  |  | |  |  |  |
|  | male | 0.02 | 0.11 | | 0.22 | 0.829 |  |
| **Highest Educational Level  (ref: Pre-U and Vocational Education )** | | | | | | |  |
|  | Polytechnic/Diploma/ Equivalent | 0.16 | 0.20 | | 0.82 | 0.412 |  |
|  | University Degree | 0.42 | 0.19 | | 2.21 | 0.028 |  |
|  | Postgraduate Degree | 0.75 | 0.21 | | 3.61 | < 0.001 |  |
| **Level of preoccupations and troubles  (ref: No Troubles/ Preoccupations)** | | | | | | |  |
|  | Minimal troubles/  preoccupations | 0.10 | 0.12 | | 0.86 | 0.392 |  |
|  | Some troubles/  preoccupations | 0.02 | 0.12 | | 0.15 | 0.882 |  |
|  | Many troubles/  preoccupations | 0.12 | 0.20 | | 0.60 | 0.548 |  |

**Supplementary Table S4.10** Association between having been actively involved in the care of a family member /close friend and PPOS scores, adjusted for age, gender, and education.

| **Predictor** | | **Unstandardised Estimate** | **Standard Error** | ***t* statistic** | ***p*-value** |  |
| --- | --- | --- | --- | --- | --- | --- |
|  |  |  |  |  |  |  |
| **Intercept** |  | 3.49 | 0.19 | 18.15 | < 0.001 |  |
| **Age (ref: 21 - 29 years)** | |  | | | |  |
|  | 30 - 39 years | -0.04 | 0.10 | -0.43 | 0.669 |  |
|  | 40 - 49 years | 0.08 | 0.11 | 0.66 | 0.509 |  |
|  | ≥ 50 years | -0.01 | 0.14 | -0.08 | 0.932 |  |
| **Gender (ref: female)** | |  |  |  |  |  |
|  | male | 0.03 | 0.11 | 0.26 | 0.795 |  |
| **Highest Educational Level  (ref: Pre-U and Vocational Education)** | | | | | |  |
|  | Polytechnic/Diploma/ Equivalent | 0.11 | 0.19 | 0.59 | 0.555 |  |
|  | University Degree | 0.33 | 0.18 | 1.82 | 0.07 |  |
|  | Postgraduate Degree | 0.67 | 0.20 | 3.37 | < 0.001 |  |
| **Having been actively involved in the  care of a family member or close friend  (ref: No)** | | | | | |  |
|  | Yes | -0.04 | 0.08 | -0.45 | 0.652 |  |

**Supplementary Table S4.11** Association between having received any care for an acute or chronic medical condition and PPOS scores, adjusted for age, gender, and education.

| **Predictor** | | **Unstandardised Estimate** | **Standard Error** | ***t* statistic** | ***p*-value** |  |
| --- | --- | --- | --- | --- | --- | --- |
|  |  |  |  |  |  |  |
| **Intercept** |  | 3.41 | 0.19 | 17.67 | < 0.001 |  |
| **Age (ref: 21 - 29 years)** | |  | | | |  |
|  | 30 - 39 years | -0.03 | 0.10 | -0.27 | 0.789 |  |
|  | 40 - 49 years | 0.09 | 0.12 | 0.81 | 0.419 |  |
|  | ≥ 50 years | 0.02 | 0.14 | 0.16 | 0.872 |  |
| **Gender (ref: female)** | |  |  |  |  |  |
|  | male | 0.02 | 0.10 | 0.16 | 0.875 |  |
| **Highest Educational Level (ref: Pre-U and Vocational Education)** | |  | | | |  |
|  | Polytechnic/Diploma/ Equivalent | 0.09 | 0.19 | 0.49 | 0.624 |  |
|  | University Degree | 0.30 | 0.18 | 1.67 | 0.096 |  |
|  | Postgraduate Degree | 0.66 | 0.20 | 3.31 | 0.001 |  |
| **Received any care for an acute or chronic medical condition (ref: No)** | |  | | | |  |
|  | Yes | 0.10 | 0.08 | 1.31 | 0.192 |  |

**Supplementary Table S4.12** Association between the frequency of attending to the same patient over 6 months and PPOS scores, adjusted for age, gender, and education.

| **Predictor** | | **Unstandardised Estimate** | **Standard Error** | ***t* statistic** | ***p*-value** |  |
| --- | --- | --- | --- | --- | --- | --- |
|  |  |  |  |  |  |  |
| **Intercept** |  | 3.24 | 0.19 | 16.85 | < 0.001 |  |
| **Age (ref: 21 - 29 years)** | |  | | | |  |
|  | 30 - 39 years | -0.03 | 0.10 | -0.33 | 0.745 |  |
|  | 40 - 49 years | 0.10 | 0.12 | 0.90 | 0.369 |  |
|  | ≥ 50 years | 0.01 | 0.13 | 0.07 | 0.941 |  |
| **Gender (ref: female)** | |  |  |  |  |  |
|  | male | -0.01 | 0.10 | -0.13 | 0.894 |  |
| **Highest Educational Level (ref: Pre-U and Vocational Education)** | |  | | | |  |
|  | Polytechnic/Diploma/ Equivalent | 0.23 | 0.17 | 1.34 | 0.18 |  |
|  | University Degree | 0.40 | 0.16 | 2.48 | 0.014 |  |
|  | Postgraduate Degree | 0.79 | 0.18 | 4.39 | < 0.001 |  |
| **Frequency of attending to the same patient over the course of six months (ref: 1-5 times )** | |  | | | |  |
|  | 5-10 times | 0.13 | 0.46 | 0.79 | 0.429 |  |
|  | 10-20 times | 0.22 | 0.49 | 1.58 | 0.115 |  |
|  | 20-30 times | 0.26 | 0.53 | 1.95 | 0.052 |  |
|  | > 30 times | 0.03 | 0.28 | 0.27 | 0.786 |  |

**Supplementary Table S4.13** Association between the most common type of patients served and PPOS scores, adjusted for age, gender, and education.

| **Predictor** | | **Unstandardised Estimate** | **Standard Error** | ***t* statistic** | ***p*-value** |  |
| --- | --- | --- | --- | --- | --- | --- |
|  |  |  |  |  |  |  |
| **Intercept** |  | 3.38 | 0.20 | 17.27 | < 0.001 |  |
| **Age (ref: 21 - 29 years)** | |  | | | |  |
|  | 30 - 39 years | -0.06 | 0.10 | -0.65 | 0.517 |  |
|  | 40 - 49 years | 0.08 | 0.11 | 0.67 | 0.502 |  |
|  | ≥ 50 years | 0.01 | 0.13 | 0.09 | 0.928 |  |
| **Gender (ref: female)** | |  |  |  |  |  |
|  | male | 0.03 | 0.10 | 0.27 | 0.791 |  |
| **Highest Educational Level (ref: Pre-U and Vocational Education)** | |  | | | |  |
|  | Polytechnic/Diploma/ Equivalent | 0.29 | 0.18 | 1.65 | 0.101 |  |
|  | University Degree | 0.42 | 0.16 | 2.59 | 0.01 |  |
|  | Postgraduate Degree | 0.79 | 0.18 | 4.35 | < 0.001 |  |
| **Most common type of patients served (ref: Palliative Patients)** | |  | | | |  |
|  | Dementia patients | -0.05 | 0.14 | -0.37 | 0.714 |  |
|  | Rehabilitation patients | 0.03 | 0.10 | 0.30 | 0.763 |  |
|  | Wound care patients | -0.31 | 0.13 | -2.33 | 0.021 |  |
|  | Others | -0.09 | 0.23 | -0.40 | 0.69 |  |

**Supplementary Table S4.14** Association between the number of patients seen on an average day and PPOS scores, adjusted for age, gender, and education.

| **Predictor** | | **Unstandardised Estimate** | **Standard Error** | ***t* statistic** | ***p*-value** |
| --- | --- | --- | --- | --- | --- |
|  |  |  |  |  |  |
| **Intercept** |  | 3.55 | 0.17 | 20.29 | < 0.001 |
| **Age (ref: 21 - 29 years)** | |  | | | |
|  | 30 - 39 years | -0.04 | 0.10 | -0.38 | 0.708 |
|  | 40 - 49 years | 0.08 | 0.11 | 0.68 | 0.494 |
|  | ≥ 50 years | -0.01 | 0.13 | -0.10 | 0.924 |
| **Gender (ref: female)** | |  |  |  |  |
|  | male | -0.03 | 0.10 | -0.30 | 0.762 |
| **Highest Educational Level (ref: Pre-U and Vocational Education)** | |  | | | |
|  | Polytechnic/Diploma/ Equivalent | 0.29 | 0.17 | 1.74 | 0.084 |
|  | University Degree | 0.47 | 0.16 | 2.99 | 0.003 |
|  | Postgraduate Degree | 0.78 | 0.18 | 4.41 | < 0.001 |
| **Number of patients seen on an average day** | |  | | | |
|  | Number of patients seen on an average day | -0.02 | 0.01 | -2.83 | 0.005 |

**Supplementary Table S4.15** Association between the number of hours worked on an average and PPOS scores, adjusted for age, gender, and education.

| **Predictor** | | **Unstandardised Estimate** | **Standard Error** | ***t* statistic** | ***p*-value** |  |
| --- | --- | --- | --- | --- | --- | --- |
|  |  |  |  |  |  |  |
| **Intercept** |  | 3.26 | 0.21 | 15.90 | < 0.001 |  |
| **Age (ref: 21 - 29 years)** | |  | | | |  |
|  | 30 - 39 years | -0.03 | 0.10 | -0.32 | 0.75 |  |
|  | 40 - 49 years | 0.10 | 0.11 | 0.87 | 0.384 |  |
|  | ≥ 50 years | 0.03 | 0.14 | 0.23 | 0.819 |  |
| **Gender (ref: female)** | |  |  |  |  |  |
|  | male | 0.02 | 0.10 | 0.24 | 0.811 |  |
| **Highest Educational Level (ref: Pre-U and Vocational Education)** | |  | | | |  |
|  | Polytechnic/Diploma/ Equivalent | 0.18 | 0.17 | 1.09 | 0.276 |  |
|  | University Degree | 0.39 | 0.16 | 2.44 | 0.016 |  |
|  | Postgraduate Degree | 0.73 | 0.18 | 4.10 | < 0.001 |  |
| **Number of hours worked on an average week** | |  | | | |  |
|  | Number of hours worked on an average week | 0.00 | 0.00 | 0.99 | 0.325 |  |

**Supplementary Table S4.16** Association between the number of years worked in healthcare and PPOS scores, adjusted for age, gender, and education.

| **Predictor** | | **Unstandardised Estimate** | **Standard Error** | ***t* statistic** | ***p*-value** |  |
| --- | --- | --- | --- | --- | --- | --- |
|  |  |  |  |  |  |  |
| **Intercept** |  | 3.38 | 0.17 | 20.21 | < 0.001 |  |
| **Age (ref: 21 - 29 years)** | |  | | | |  |
|  | 30 - 39 years | -0.02 | 0.11 | -0.19 | 0.85 |  |
|  | 40 - 49 years | 0.14 | 0.14 | 1.05 | 0.295 |  |
|  | ≥ 50 years | 0.08 | 0.18 | 0.46 | 0.644 |  |
| **Gender (ref: female)** | |  |  |  |  |  |
|  | male | 0.01 | 0.10 | 0.09 | 0.928 |  |
| **Highest Educational Level (ref: Pre-U and Vocational Education )** | |  | | | |  |
|  | Polytechnic/Diploma/ Equivalent | 0.22 | 0.17 | 1.28 | 0.202 |  |
|  | University Degree | 0.42 | 0.16 | 2.61 | 0.01 |  |
|  | Postgraduate Degree | 0.78 | 0.18 | 4.26 | < 0.001 |  |
| **Years worked in healthcare** | |  | | | |  |
|  | Years worked in healthcare | 0.00 | 0.01 | -0.71 | 0.478 |  |

**Supplementary Table S4.17** Association between professional role and PPOS scores, adjusted for age, gender, and education.

| **Predictor** | | **Unstandardised Estimate** | **Standard Error** | ***t* statistic** | ***p*-value** |  |
| --- | --- | --- | --- | --- | --- | --- |
|  |  |  |  |  |  |  |
| **Intercept** |  | 3.90 | 0.22 | 18.08 | < 0.001 |  |
| **Age (ref: 21 - 29 years)** | |  | | | |  |
|  | 30 - 39 years | 0.04 | 0.10 | 0.39 | 0.698 |  |
|  | 40 - 49 years | 0.13 | 0.11 | 1.19 | 0.237 |  |
|  | ≥ 50 years | -0.02 | 0.13 | -0.14 | 0.886 |  |
| **Gender (ref: female)** | |  |  |  |  |  |
|  | male | -0.13 | 0.10 | -1.28 | 0.201 |  |
| **Highest Educational Level (ref: Pre-U and Vocational Education )** | |  | | | |  |
|  | Polytechnic/Diploma/ Equivalent | 0.33 | 0.17 | 1.96 | 0.052 |  |
|  | University Degree | 0.35 | 0.17 | 2.09 | 0.037 |  |
|  | Postgraduate Degree | 0.57 | 0.19 | 2.92 | 0.004 |  |
| **Professional Role (ref: Physician)** | |  | | | |  |
|  | Junior Nursing Staff^a^ | -0.89 | 0.15 | -5.83 | < 0.001 |  |
|  | Senior Nursing Staff* | -0.58 | 0.15 | -3.87 | < 0.001 |  |
|  | Therapist | -0.23 | 0.15 | -1.52 | 0.13 |  |
|  | Therapy Aide | -0.37 | 0.18 | -1.99 | 0.047 |  |

*note:
a Junior Nursing Staff refer to nursing aides, enrolled nurses and senior enrolled nurses. Senior Nursing Staff refer to staff nurses, senior staff nurses, advanced practitioner nurses and nursing managers.*

**Supplementary Table S4.18** Association between employment status and PPOS scores, adjusted for age, gender, and education.

| **Predictor** | | **Unstandardised Estimate** | **Standard Error** | ***t* statistic** | ***p*-value** |  |
| --- | --- | --- | --- | --- | --- | --- |
|  |  |  |  |  |  |  |
| **Intercept** |  | 3.32 | 0.17 | 19.21 | < 0.001 |  |
| **Age (ref: 21 - 29 years)** | |  | | | |  |
|  | 30 - 39 years | -0.05 | 0.10 | -0.49 | 0.627 |  |
|  | 40 - 49 years | 0.08 | 0.11 | 0.71 | 0.48 |  |
|  | ≥ 50 years | -0.03 | 0.14 | -0.22 | 0.825 |  |
| **Gender (ref: female)** | |  |  |  |  |  |
|  | male | 0.02 | 0.10 | 0.21 | 0.832 |  |
| **Highest Educational Level (ref: Pre-U and Vocational Education )** | |  | | | |  |
|  | Polytechnic/Diploma/ Equivalent | 0.26 | 0.17 | 1.49 | 0.137 |  |
|  | University Degree | 0.45 | 0.16 | 2.76 | 0.006 |  |
|  | Postgraduate Degree | 0.79 | 0.18 | 4.38 | < 0.001 |  |
| **Employment status (ref: Employed Full-time)** | |  | | | |  |
|  | Employed Part-time | 0.21 | 0.17 | 1.25 | 0.214 |  |

## Supplementary Section S5: Multiple Linear Regression: Model Comparisons and Model 2

**Supplementary Table S5.1:** Comparison of Multiple Linear Regression Models Predicting PPOS Scores

| **Model** | **R^2^** | **Adjusted R^2^** | **AIC** | **BIC** | **F-statistic** | **df1** | **df2** |
| --- | --- | --- | --- | --- | --- | --- | --- |
| **Model 2 (Full)** | 0.48 | 0.31 | 396 | 588 | 2.90 | 54 | 170 |
| **Model 3 (Final)** | 0.37 | 0.30 | 430 | 526 | 5.49 | 25 | 235 |

**Supplementary Table S5.2** Multiple Linear Regression Model 2 showing associations between individual, professional, and organisational factors and patient-centred orientation (PPOS scores), adjusted for age, gender, and highest educational level.

| **Variable** | | **Estimate** | **SE** | ***t* statistic** | ***p-*value** |
| --- | --- | --- | --- | --- | --- |
| **Intercept** |  | 4.10 | 0.80 | 5.10 | <.001 |
| **Age (ref: 21-29 years)** | |  | | | |
|  | 30-39 years | 0.00 | 0.12 | 0.00 | 0.999 |
|  | 40-49 years | 0.07 | 0.15 | 0.45 | 0.657 |
|  | ≥50 years | 0.04 | 0.20 | 0.19 | 0.846 |
| **Gender (ref: female)** | |  | | | |
|  | Male | -0.06 | 0.11 | -0.52 | 0.607 |
| **Educational Level (ref: Pre-U and Vocational Education )** | |  | | | |
|  | Polytechnic/ Diploma | 0.34 | 0.25 | 1.35 | 0.179 |
|  | University Degree | 0.32 | 0.24 | 1.34 | 0.182 |
|  | Postgraduate Degree | 0.50 | 0.27 | 1.86 | 0.065 |
| **Hospital Culture Promotes Patient Centred Care (ref: Not at all/To a small extent)** | |  | | | |
|  | To some extent | 0.44 | 0.21 | 2.09 | 0.038 |
|  | To a great extent | 0.62 | 0.20 | 3.03 | 0.003 |
|  | Completely | 0.62 | 0.22 | 2.79 | 0.006 |
| **Employment Status (ref: Full-time)** | |  | | | |
|  | Part-time | 0.10 | 0.18 | 0.55 | 0.58 |
| **Current Role (ref: Physician)** | |  | | | |
|  | Junior Nursing Staff^a^ | -0.62 | 0.19 | -3.25 | 0.001 |
|  | Senior Nursing Staff^a^ | -0.50 | 0.18 | -2.77 | 0.006 |
|  | Therapist | -0.10 | 0.21 | -0.50 | 0.616 |
|  | Therapy Aide | -0.39 | 0.25 | -1.55 | 0.122 |
| **Years worked in healthcare** | | -0.01 | 0.01 | -0.91 | 0.365 |
| **Number of hours worked on an average week** | | 0.00 | 0.00 | 0.42 | 0.672 |
| **Number of patients seen on an average day** | | 0.00 | 0.01 | -0.08 | 0.934 |
| **Most common type of patients served (ref: Palliative patients)** | |  | | | |
|  | Dementia patients | -0.12 | 0.15 | -0.85 | 0.397 |
|  | Rehabilitation patients | -0.14 | 0.12 | -1.18 | 0.239 |
|  | Wound care patients | -0.25 | 0.14 | -1.75 | 0.081 |
|  | Others | 0.06 | 0.30 | 0.20 | 0.838 |
| **Frequency of attending to the same patient over the course of six months (ref: 1-5 times)** | |  | | | |
|  | 5-10 times | 0.09 | 0.18 | 0.48 | 0.631 |
|  | 10-20 times | 0.12 | 0.15 | 0.78 | 0.437 |
|  | 20-30 times | 0.25 | 0.16 | 1.59 | 0.114 |
|  | > 30 times | 0.05 | 0.14 | 0.39 | 0.7 |
| **Received any care for an acute or chronic medical condition (ref: No)** | |  | | | |
|  | Yes | 0.08 | 0.08 | 0.95 | 0.343 |
| **Been actively involved in the care of a family member or close friend (ref: No)** | |  | | | |
|  | Yes | -0.10 | 0.09 | -1.17 | 0.243 |
| **Level of preoccupations and troubles in one's private life (ref: None)** | |  | | | |
|  | Minimal preoccupations | 0.05 | 0.12 | 0.39 | 0.7 |
|  | Some preoccupations | 0.00 | 0.13 | 0.04 | 0.972 |
|  | Many preoccupations | -0.17 | 0.22 | -0.75 | 0.455 |
| **Frequency of cultural barriers during patient interactions (ref: Not at all)** | |  | | | |
|  | Rarely | -0.20 | 0.21 | -0.96 | 0.34 |
|  | Sometimes | -0.27 | 0.21 | -1.25 | 0.212 |
|  | Always | 0.21 | 0.29 | 0.74 | 0.462 |
| **Frequency of language barriers during patient interactions (ref: Not at all)** | |  | | | |
|  | Rarely | -0.28 | 0.44 | -0.64 | 0.522 |
|  | Sometimes | -0.31 | 0.44 | -0.70 | 0.482 |
|  | Frequently | -0.17 | 0.45 | -0.38 | 0.702 |
|  | Always | 0.13 | 0.53 | 0.24 | 0.81 |
| **Support at work for difficult patient interactions**  **(ref: Not at all/ Small extent)** | |  | | | |
|  | To some extent | -0.24 | 0.20 | -1.20 | 0.232 |
|  | To a great extent | -0.10 | 0.20 | -0.49 | 0.626 |
|  | Completely | -0.13 | 0.23 | -0.58 | 0.562 |
| **Work is meaningful (ref: Disagree/ Neutral)** | |  | | | |
|  | Agree | 0.05 | 0.20 | 0.24 | 0.813 |
|  | Strongly Agree | 0.25 | 0.22 | 1.13 | 0.262 |
| **Work is satisfying (ref: Strongly Disagree/ Disagree)** | |  | | | |
|  | Neutral | -0.14 | 0.35 | -0.41 | 0.679 |
|  | Agree | 0.10 | 0.34 | 0.29 | 0.775 |
|  | Strongly Agree | -0.05 | 0.35 | -0.15 | 0.877 |
| **Workload is manageable (ref: Strongly Disagree)** | |  | | | |
|  | Disagree | -0.59 | 0.31 | -1.89 | 0.061 |
|  | Neutral | -0.49 | 0.30 | -1.61 | 0.109 |
|  | Agree | -0.64 | 0.31 | -2.10 | 0.038 |
|  | Strongly Agree | -0.52 | 0.34 | -1.54 | 0.125 |
| **Level of Burnout^b^ (ref: 1)** | |  | | | |
|  | 2 | 0.37 | 0.14 | 2.63 | 0.009 |
|  | 3 | 0.16 | 0.17 | 0.99 | 0.322 |
|  | 4 | 0.10 | 0.24 | 0.43 | 0.667 |
|  | 5 | 0.21 | 0.31 | 0.68 | 0.496 |

*note:*

*a Junior Nursing Staff refer to nursing aides, enrolled nurses and senior enrolled nurses. Senior Nursing Staff refer to staff nurses, senior staff nurses, advanced practitioner nurses and nursing managers.*

*b Level of burnout was assessed using the Single Item Burnout Measure, with responses on an ordinal scale: 1= I enjoy my work. I have no symptoms of burnout; 2= Occasionally I am under stress, and I don’t always have as much energy as I once did, but I don’t feel burned out; 3= I am definitely burning out and have one or more symptoms of burnout, such as physical and emotional exhaustion; 4=The symptoms of burnout that I’m experiencing won’t go away. I think about frustration at work a lot; 5= I feel completely burned out and often wonder if I can go on. I am at the point where I may need some changes or may need to seek some sort of help.*

## Supplementary Section S6: Questionnaire and patient information sheet and consent form

**CONSENT FORM (Online format)**

**Purpose of the Study:** You are invited to participate in a research study examining the healthcare provision styles of healthcare professionals in St Luke’s Hospital. You must be 21 years and above, and working as a doctor, nurse or rehabilitation therapist at St Luke’s Hospital.

**Procedures:** If you choose to participate, you will answer some questions about your sociodemographic information, occupation, professional experience and attitudes towards patient-centredness. The survey is expected to take approximately 10 minutes. You may only submit the survey once.

Please read the questions carefully and answer all questions truthfully.

**Voluntary Participation:** Your participation in this research study is voluntary and you

may choose to not participate or end your participation at any time without penalty

**Confidentiality:** The data collected in this study are completely **anonymous**. No personally identifiable information will be collected and the information you choose to provide in this study cannot be connected back to you. Results from this study may be published or presented at research conferences.

**Possible Risks and Benefits:** It is unlikely that you will experience any risks or discomforts

beyond what would be experienced in everyday life by participating. There are no specific

benefits associated with participating

**Who to Contact if You Have Questions or Concerns**

The study has been reviewed by the Institutional Review Board (the hospital’s research ethics committee) for ethics approval.

If you have questions about this research study, you may contact the Principal Investigator:
Dr Chiong Xiao-Ying, Cleo

Medical Department, St Luke’s Hospital

Tel: 98301880 (Mobile) 68952792 (Office) or email: cleochiongxy@stluke.org.sg

If you have any complaints or feedback about this study, you may contact the Principal Investigator or the Institutional Review Board Secretariat (irbs@stluke.org.sg)

**Consent**: I have read and understand the above consent form. I certify that I am 21 years old or older. By clicking the “Next” button to enter the survey, I indicate my willingness to voluntarily take part in this study.

**Section A: Participant characteristics**

1. Age

- 21 - 29 years
- 30 – 39 years
- 40 – 49 years
- ≥ 50 years

2. Gender

- Male
- Female

3. What is your highest education level completed?

- Secondary School (O’ or N’ levels/Equivalent)
- Junior College (A’ levels/Equivalent)
- ITE/Normal Technical Certificate/Equivalent
- Polytechnic/Diploma/Equivalent
- University Degree
- Post-graduate Degree

4. Employment status:

- Employed, full-time
- Employed part-time

5. Current role:

- Physician
- Nursing Aide / Enrolled Nurse / Senior Enrolled Nurse
- Staff Nurse / Senior staff nurse/ Advanced Practitioner Nurse/ Nursing Manager
- Physiotherapist / Occupational / Speech therapist/ Art therapist / Music therapist
- Therapy Aide (PT/ OT/ MT/ AT)

6. How many years have you been working in healthcare?  ___ years

7. How many hours do you work on an average week? _____
8. How many patients do you see on an average day? ______
9. What is the most common type of patients you serve?

- Palliative patients
- Dementia patients
- Rehabilitation patients
- Cancer patients
- Wound care patients
- Others (please specify) :______

10. Have you ever received any care for an acute or chronic medical condition from a physician, nurse, or allied health professional?

- Yes
- No
- Do not want to answer

11. Have you ever been actively involved in the care of a family member or close friend (e.g. consultation with healthcare professionals, care-related decision making, helping the person follow their treatment)?

- Yes
- No
- Do not want to answer

12. How would you describe your current level of personal troubles or preoccupations in your private life?

- No troubles/ preoccupations
- Minimal troubles/ preoccupations
- Some troubles/ preoccupations
- Many troubles/ preoccupations
- Extremely high amount of troubles/ preoccupations
- Do not want to answer

**Section B: Professional Experience and Patient interaction**

13. How often do you face cultural barriers when interacting with patients?

- Not at all
- Rarely
- Sometimes
- Frequently
- Always

14. How often do you face language barriers when interacting with patients?

- Not at all
- Rarely
- Sometimes
- Frequently
- Always

15. Do you feel adequately supported by your colleagues or supervisors when dealing with difficult patient interactions?

- Not at all
- To a small extent
- To some extent
- To a great extent
- Completely

16. The work that I do is meaningful.

- Strongly disagree
- Disagree
- Neutral
- Agree
- Strongly agree

17. My job is overall satisfying.

- Strongly disagree
- Disagree
- Neutral
- Agree
- Strongly agree

18. My overall workload is manageable within my working hours.

- Strongly disagree
- Disagree
- Neutral
- Agree
- Strongly agree

19. Overall, based on your definition of burnout, how would you rate your level of burnout?

- I enjoy my work. I have no symptoms of burnout.
- Occasionally I am under stress, and I don’t always have as much energy as I once did, but I don’t feel burned out.
- I am definitely burning out and have one or more symptoms of burnout, such as physical and emotional exhaustion.
- The symptoms of burnout that I’m experiencing won’t go away. I think about frustration at work a lot.
- I feel completely burned out and often wonder if I can go on. I am at the point where I may need some changes or may need to seek some sort of help.

20. To what extent does your hospital culture promote patient-centred care?
    *(Patient-centred care is a healthcare approach that prioritizes the needs, preferences, and values of each patient, actively involving patients in decisions about their care.)*

- Not at all
- To a small extent
- To some extent
- To a great extent
- Completely

**Section C: Patient-Practitioner Orientation Scale**

(Reproduced with permission from Ed Krupat, Harvard Medical School)

The statements below refer to beliefs that people might have concerning healthcare professionals, patients, and care. Read each item and indicate how much you agree or disagree with each.

Definitions of terms used in this scale:
Health-care professional (HCP) = Yourself (doctor/nurse/therapist)
Care encounter = an interaction between yourself and a patient for the purpose of providing care

1. The healthcare professional is the one who should decide what gets talked about during a care encounter.

- Strongly disagree
- Moderately disagree
- Slightly disagree
- Slightly agree
- Moderately agree
- Strongly agree

2. Healthcare doesn’t feel as personal these days, but this is a small sacrifice for helpful new tools like video calls with doctors, apps to send blood pressure readings, and chatbots for advice.

- Strongly disagree
- Moderately disagree
- Slightly disagree
- Slightly agree
- Moderately agree
- Strongly agree

3. The most important part of the standard care encounter is the healthcare professional’s assessment.

- Strongly disagree
- Moderately disagree
- Slightly disagree
- Slightly agree
- Moderately agree
- Strongly agree

4. It is often best for patients if they do not have a full explanation of their medical condition.

- Strongly disagree
- Moderately disagree
- Slightly disagree
- Slightly agree
- Moderately agree
- Strongly agree

5. Patients should rely on their healthcare professional’s knowledge and not try to find out about their conditions on their own.

- Strongly disagree
- Moderately disagree
- Slightly disagree
- Slightly agree
- Moderately agree
- Strongly agree

6. When healthcare professionals ask a lot of questions about a patient’s background, they are prying too much into personal matters.

- Strongly disagree
- Moderately disagree
- Slightly disagree
- Slightly agree
- Moderately agree
- Strongly agree

7. If healthcare professionals are truly good at diagnosis and/or treatment, the way they relate to patients is not that important.

- Strongly disagree
- Moderately disagree
- Slightly disagree
- Slightly agree
- Moderately agree
- Strongly agree

8. Many patients continue asking questions even though they are not learning anything new by doing so.

- Strongly disagree
- Moderately disagree
- Slightly disagree
- Slightly agree
- Moderately agree
- Strongly agree

9. Patients should be treated as if they were partners with the healthcare professional, equal in power and status.

- Strongly disagree
- Moderately disagree
- Slightly disagree
- Slightly agree
- Moderately agree
- Strongly agree

10. Patients generally want reassurance rather than information about their health.

- Strongly disagree
- Moderately disagree
- Slightly disagree
- Slightly agree
- Moderately agree
- Strongly agree

11. If a healthcare professional’s primary tools are being open and warm, the healthcare professional will not have a lot of success.

- Strongly disagree
- Moderately disagree
- Slightly disagree
- Slightly agree
- Moderately agree
- Strongly agree

12. When patients disagree with their healthcare professional, this is a sign that the healthcare professional does not have the patient’s respect and trust.

- Strongly disagree
- Moderately disagree
- Slightly disagree
- Slightly agree
- Moderately agree
- Strongly agree

13. A treatment plan cannot succeed if it is in conflict with a patient’s lifestyle or values.

- Strongly disagree
- Moderately disagree
- Slightly disagree
- Slightly agree
- Moderately agree
- Strongly agree

14. Most patients want to end the care encounter with their healthcare professional as quickly as possible. (e.g. in the clinic, they would want to leave the consult quickly; in the ward, they would like to end their therapy or nursing encounter quickly).

- Strongly disagree
- Moderately disagree
- Slightly disagree
- Slightly agree
- Moderately agree
- Strongly agree

15. The patient must always be aware that the healthcare professional is in charge.

- Strongly disagree
- Moderately disagree
- Slightly disagree
- Slightly agree
- Moderately agree
- Strongly agree

16. Knowing a patient’s culture or background is not necessary to treat or manage their illness well.

- Strongly disagree
- Moderately disagree
- Slightly disagree
- Slightly agree
- Moderately agree
- Strongly agree

17. Humor is a major ingredient in the care of the patient.

- Strongly disagree
- Moderately disagree
- Slightly disagree
- Slightly agree
- Moderately agree
- Strongly agree

18. When patients look up medical information on their own, this usually confuses more than it helps.

- Strongly disagree
- Moderately disagree
- Slightly disagree
- Slightly agree
- Moderately agree
- Strongly agree
